# Gene–Dairy Food Interactions and Health Outcomes: A Review of Nutrigenetic Studies

**DOI:** 10.3390/nu9070710

**Published:** 2017-07-06

**Authors:** Kevin B. Comerford, Gonca Pasin

**Affiliations:** California Dairy Research Foundation (CDRF), 501 G Street, Ste. 203, Davis, CA 95616, USA; pasin@cdrf.org

**Keywords:** nutrigenetics, gene–diet interactions, precision nutrition, polymorphisms, inter-individual response, dairy, milk, lactase persistence, obesity, cardiometabolic disease

## Abstract

Each person differs from the next by an average of over 3 million genetic variations in their DNA. This genetic diversity is responsible for many of the interindividual differences in food preferences, nutritional needs, and dietary responses between humans. The field of nutrigenetics aims to utilize this type of genetic information in order to personalize diets for optimal health. One of the most well-studied genetic variants affecting human dietary patterns and health is the lactase persistence mutation, which enables an individual to digest milk sugar into adulthood. Lactase persistence is one of the most influential Mendelian factors affecting human dietary patterns to occur since the beginning of the Neolithic Revolution. However, the lactase persistence mutation is only one of many mutations that can influence the relationship between dairy intake and disease risk. The purpose of this review is to summarize the available nutrigenetic literature investigating the relationships between genetics, dairy intake, and health outcomes. Nonetheless, the understanding of an individual’s nutrigenetic responses is just one component of personalized nutrition. In addition to nutrigenetic responses, future studies should also take into account nutrigenomic responses (epigenomic, transcriptomic, proteomic, metabolomic), and phenotypic/characteristic traits (age, gender, activity level, disease status, etc.), as these factors all interact with diet to influence health.

## 1. Introduction

The fields of nutrigenetics and nutrigenomics both aim to elucidate how the genome interacts with nutrition to influence health. They differ in that nutrigenetics focuses on how gene variants influence responses to diet, while nutrigenomics focuses on how diet affects gene expression. The intended end goals of these sciences, which both fall under the umbrella of nutritional genomics, are to improve health through the application of personalized nutrition [1]. At present, most of the well-studied nutritional genomic relationships are nutrigenetic. More specifically, the most well-known examples can all be classified as monogenic relationships, consisting of the interactions between a single gene mutation and single dietary component. For example, a mutation in the gene coding for the enzyme phenylalanine hydroxylase leads to an inability to properly metabolize the amino acid phenylalanine, resulting in the life-threatening disease phenylketonuria (PKU) [2]. A mutation in the alcohol dehydrogenase gene leads to a reduced ability to process alcohol and has been associated with addiction [3]. Mutations in the 5,10-methylene tetrahydrofolate reductase (MTHFR) gene affect folate metabolism and are linked to increased risk for birth defects and chronic diseases including cardiovascular disease (CVD) and cancer [4]. Mutations in the lactase gene (LCT) can result in lactase persistence (LP), which is the extended ability to produce the lactase enzyme into adulthood. Lactase assists in the breakdown of milk sugar in the small intestine and is associated with a variety of complex health and disease outcomes in different populations [5,6].

The vast majority of genetic variants that have nutrigenetic consequences are loss of function mutations in which the ability to properly digest or metabolize dietary compounds are diminished. LP stands apart from the majority of monogenic mutations in that it is a gain of function mutation in which the ability to break down lactose into adulthood is gained. Nutrigenetic loss of function mutations tend to restrict or reduce dietary options, but LP does the opposite and increases the available number of dietary options that can be digested. This ability has resulted in LP becoming one of the strongest positive selective pressures in human history [7], reinforcing the importance of gene–dairy food interactions in relation to human reproductive success and survival [8].

LP polymorphisms have occurred independently in Northern Europe, Africa and the Middle East. This dominant trait provided these populations with the ability to greatly expand their cuisines and improve their access to energy, nutrients, and dietary sources of potable water [9,10]. LP gene variants have also dramatically influenced gene–culture co-evolution between humans and cattle through the practices of dairy herding, domestication, and farming [7,11]. Many of the implications of these interrelationships have yet to be discovered. While LP mutations have been advantageous for the survival of certain populations over the last several millennia, only recently have the scientific tools become available to investigate how the nutrigenetic relationships between gene variants and dairy intake affect human health. The purpose of this review is to summarize the available nutrigenetic literature investigating the relationships between genetics, dairy intake, and health outcomes in order to further explore the concept of personalized nutrition. The PubMed database was searched through the end of April 2017 for studies that examined the relationships between genetic variants, dairy intake, and health outcomes. We only included studies that reported at least one measure of dairy intake. Seventeen studies on LP polymorphisms and nine studies on metabolic and hormonal gene variants were included in the review and summarized in table form.

## 2. Lactase Persistence, Dairy Intake, and Health Outcomes

The LCT-13910 C > T single nucleotide polymorphism (SNP) at intron 13 is the most prevalent and most studied LP polymorphism. Traditionally, a person with two CC alleles is considered to be lactose intolerant, also known as lactase non-persistent (lactase activity <10 units/g protein) [12]. A majority of humans, especially those without Northern European ancestry, have the CC genotype. A person with one or more T alleles (CT or TT) is considered to be lactose tolerant, also known as lactase persistent (lactase activity ≥10 units/g protein). Recent evidence suggests that there is a stepwise relationship between the number of LP dominant T alleles a person inherits and their ability to digest lactose [13]. However, the LCT-13910 gene variant is not the only variant that can confer LP. Several mutations at different loci on the lactase gene (i.e., A-22018, C-14010, G-13907, and G-13915) result in an LP phenotype [14]. It is currently unclear as to if and how these different gene variants affect lactase form and function. Future research on how different LP variants, LP genotypes, and LP haplotypes interact with dairy intake patterns and health outcomes would contribute greatly to the basic understanding of gene–dairy interactions. However, at present, the available nutrigenetic research on LP, dairy intake, and health is still in the early stages and limited in its ability to inform personalized dietary advice (Table 1).

### 2.1. Body Mass Index (BMI), Overweight, Obesity

The study of nutrigenetics is expanding rapidly as researchers are now investigating the polygenic nature of several disease states. In fact, only a small percentage of obesity cases have been shown to be monogenic [30]. Rather, inherited predispositions to obesity are primarily polygenic and involve both dietary and non-dietary factors [30,31]. However, if a researcher decided to select only a single gene mutation to study in relation to body weight, the LP mutation might be a good candidate to interrogate due to its strong selective advantage and frequency of occurrence in countries with high obesity rates. In fact, several studies have found correlations between LP and higher body mass index (BMI) [32,33]. However, many of these studies use LP as a proxy for dairy intake rather than actually measuring or recording dairy intake. At first glance, using LP as a proxy for milk or dairy intake appears to be a common and reasonable assumption to make for European populations. Yet, this proxy may not actually represent what any particular individual in the population is consuming since dairy intake and LP prevalence both vary considerably from one European country to the next [13]. Furthermore, LP may not be an accurate proxy for overall dairy intake in most countries since many low-lactose and fermented dairy products are consumed by individuals who are lactose intolerant [34]. For this research paper, we have chosen to focus on studies that have included both LP genotype data and milk or dairy intake data, rather than rely solely on data that used LP as a proxy milk or dairy intake.

In agreement with the epidemiological data, several nutrigenetic studies of mixed design which did measure dairy intake also found the LCT-13910 C > T SNP to be correlated with higher dairy intake and BMI [16,18,19,22]. Most of these studies were relatively small, ranging in size from 551 to 3575 participants. In contrast to these findings, a recent Mendelian randomization study consisting of over 97,000 participants found no evidence linking the LP mutation and dairy intake to overweight or obesity measures [20]. These conflicting results on LP, dairy intake, and body weight offer further evidence that there are many additional variables, such as other gene variants, other dietary components, lifestyle factors, and environmental factors that may interact to influence the relationship between LP and body weight. Although a single gene mutation resulting in a phenotype such as LP can have significant effects on dietary patterns, the nutrigenetic effects of that mutation will differ considerably based on the types, amounts, frequencies, and combinations of foods consumed. Dairy is a complex food group, which contains many products that differ in their macronutrient, micronutrient, and bioactive compound composition. Several of these nutrients and bioactives may protect against chronic disease independently of any effects on body weight [35,36]. Additionally, the lactose content of dairy foods differs considerably depending on the product consumed. A closer look at how different dairy foods interact with different genetic mutations is the logical next step in better understanding the relationships between genetics, dairy intake, and body weight.

### 2.2. Cardiometabolic Disease

Overweight and obesity increase the risk for cardiometabolic disorders such as metabolic syndrome, CVD, and type 2 diabetes (T2D). The majority of dairy intake studies have consistently provided evidence of neutral or protective associations related to metabolic syndrome risk factors (e.g., glucose intolerance, dyslipidemia, and hypertension) and chronic diseases such as CVD and T2D [37,38,39,40,41]. These findings suggest that dairy intake may favorably affect cardiometabolic risk in certain populations despite augmenting BMI, or possibly, that dairy-associated gains in BMI may also involve gains in lean mass or alterations to gut bacteria that are associated with reduced cardiometabolic risk factors [42,43,44].

A study of 551 participants of European descent found that the LCT-13910 C > T polymorphism in combination with higher dairy intake could increase the risk for metabolic syndrome, but only in women [17]. These findings suggest that other major genetic factors such as gender can moderate the relationships between dietary intake and cardiometabolic risk factors to greater extent than LP. Research on over 2100 healthy adults from Finland found no associations between LP, dairy intake, and multiple measures of coronary artery disease (carotid intima-media thickness, carotid artery compliance, and brachial artery flow-mediated dilation) [15]. The findings did not differ based on gender, but the subjects who were lactose intolerant consumed significantly more alcohol (and less milk) than those who were lactose tolerant, suggesting that non-dairy factors (e.g., alcohol consumption) which can affect health may also be influenced by LP status. Moderate alcohol intake can beneficially influence cardiovascular health, but excessive intake is linked to an array of cardiovascular disease states [45].

A Mendelian randomization study of over 20,000 participants from multiple racial and ethnic groups including American (Hispanics, African-American and Whites) and Spanish populations assessed the relationship between a different and less commonly studied LP polymorphism (the MCM6-rs3754686 polymorphism at intron 15). The researchers found no associations between LP and CVD or total mortality rates [23]. The researchers also reported a lack of associations between milk intake and lipid levels, CVD, or mortality rates. A Mendelian randomization meta-analysis of 32 studies involving over 197,000 adults from Europe, the US, and Australia found that LP genotype and dairy consumption were not associated with systolic blood pressure or risk of hypertension [24]. Observational studies from this meta-analysis did show that each additional serving of dairy per day was associated with lower systolic blood pressure, but these findings were not confirmed by the intervention studies included in the report, which showed no relationship between dairy intake and systolic blood pressure or hypertension. Similarly, a Mendelian randomization study of over 98,000 Danish adults which followed its participants for over five years, found no associations between the LCT-13910 C > T polymorphism or milk intake with ischemic heart disease, myocardial infarction, or any major cardiovascular risk factor measured (i.e., total cholesterol, LDL, HDL, triglycerides, or blood pressure) [21]. The same researchers also did not find any associations between the LCT-13910 C > T polymorphism or milk intake with T2D [20]. However, the researchers did note that LP subjects who did not consume milk had a slightly higher risk for T2D compared to those LP subjects who habitually consumed milk, suggesting that an LP genotype may be able to affect health independently of dairy intake. No mechanistic explanations were reported for this finding; rather the researchers suggested the relationship between abstinence from milk intake, LP status, and T2D could be potentially explained by selection bias in the study instead of biological differences between the study participants. Another Mendelian randomization study which meta-analyzed genetic data from several studies comprised of mixed populations also investigated the relationship between the LCT-13910 C > T polymorphism, milk intake, and cardiometabolic disease [29]. This study confirmed the results from earlier studies, in that the researchers reported a lack of associations between LP status, milk intake, and cardiometabolic disease (i.e., ischemic heart disease and T2D).

### 2.3. Cancer

Epidemiological studies have provided mixed results regarding dairy intake and cancer risk [46,47,48,49,50], but these relationships begin to become clearer when specific dairy products, LP gene variants, and different types of cancer are considered. For example, colorectal cancer is one of the types of cancer most heavily influenced by dietary patterns since colorectal tissue comes into direct and prolonged contact with dietary components and products of digestion on a consistent basis. The colon also contains the majority of the gut microbiota that can modulate colonic exposure to carcinogenic toxins.

Large prospective cohort studies and meta-analyses provide evidence for protective associations of dairy intake on colorectal cancer that often depend on the type and amount of dairy products consumed [49,51,52]; however, these types of studies do not usually take into account LP status. Importantly, LP status is a major factor determining the type and amount of dairy products consumed as well as the dairy-derived end-products that are exposed to the colorectal tissue (i.e., lactose moves into the large intestine in lactose intolerant individuals, while lactose is broken down to glucose and galactose and absorbed in the small intestine in lactose tolerant individuals). A nutrigenetic meta-analysis of 80 studies regarding dairy intake and colorectal cancer showed key differences are dependent on dairy intake amount and LP status [25]. In fact, the researchers reported a protective role of higher dairy intake in both high LP prevalent and high non-LP prevalent populations. In other words, this research suggests that the potential anti-colorectal cancer benefits associated with dairy intake may not be limited to those who can digest lactose. Rather, higher lactose intakes in lactose intolerant individuals may lead to the production of favorable substrates that can be used by protective colonic microflora.

Prostate cancer is one of the most common cancers in men [12]. In contrast to the colorectal tissues, the prostate does not come into direct contact with the gut microbiota or gastrointestinal contents. Several indirect mechanisms have been proposed for how diet can affect cell replication and cancer risk. One hypothesis is that the circulating lactose breakdown products glucose and galactose, which are generally higher in LP dairy consumers, may affect cancer development or progression in certain tissues [53,54]. A Swedish case-cohort study (1499 cases and 1130 controls) which tested the associations between LP status, overall dairy intake, and prostate carcinoma, reported no associations between these variables [12]. The researchers did find an association between low-fat milk intake and prostate cancer risk, but this relationship did not appear to be influenced by LP status. When LP status, dairy intake, and prostate cancer risk were assessed in the European Prospective Investigation into Cancer and Nutrition (EPIC) study, the researchers found that both LP genotypes (CT and TT) and higher dairy intake were not associated with prostate cancer risk [13].

Relatively few nutrigenetic studies have been conducted on LP status, dairy intake, and cancer types residing outside of the digestive or reproductive systems. A recent study in nearly 23,000 Swedish adults with lactose intolerance found a decreased risk of lung, breast, and ovarian cancers compared to individuals who were lactose tolerant [55]. The authors of this research hypothesize that the decreased cancer risk in lactose intolerant individuals could be associated with lower intakes of: (1) lactose or lactose-containing products; (2) various non-lactose components of dairy, and/or (3) other protective foods and beverages consumed in place of dairy foods. Unfortunately, dietary intakes were not recorded for this study, so it is not possible to determine their effects on cancer risk. Furthermore, the study design used symptomology rather than genotype to diagnose lactose intolerance, so it is also not clear as to what percentage of individuals in this study were genetically considered lactase non-persistent. A Mendelian randomization study of nearly 1100 Eastern European and Russian adults that did take into account milk intake and LP genotype found that milk intake was associated with a slightly higher risk for renal cell carcinoma, but LP genotype was not [26]. The researchers of this study concluded that it is wise to exercise caution when interpreting observational associations between milk consumption and cancer risk since any perceived relationships between the two may be due to confounding by other dietary and lifestyle factors.

### 2.4. Bone Health

Total dairy intake [56], dairy protein intake [57,58], and dairy mineral intake [59,60,61] are all associated with bone health markers such as bone mineral density (BMD) and bone turnover. The relationship between lactose intake and bone health is a little less clear. Lactose intake has not been directly associated with bone strength or structure, but the ability to digest lactose is associated with higher intake of overall dairy intake, dairy protein intake, and dairy mineral intake—all factors consistently linked to improvements in BMD and reduced bone turnover. In agreement with this concept, lactose intolerance can increase the risk for osteoporosis and bone fractures in certain populations [62].

Researchers investigating the relationships between milk intake, LP status, and bone health in 258 postmenopausal women from Austria found that LP genotype influenced BMD and bone fractures [27]. The CC genotype, which is associated with lactose intolerance/lactase non-persistence was associated with the lowest hip and spine BMD measures and highest fracture rate. The CT genotype, which confers an intermediate level of lactose tolerance/lactase persistence, was associated with lower BMD scores compared to the TT genotype (which confers the greatest level of lactose tolerance/lactase persistence). The researchers suggested that the improved bone integrity found in LP individuals was not due to higher calcium intake alone, but rather due to the additive effects of calcium and other dairy components such as dairy proteins. In contrast to these findings, a cross-sectional study of two postmenopausal cohorts from Northern Europe found no differences in adjusted BMD or in fracture incidence between lactose tolerant and lactose intolerant women [28]. The researchers of this study came to very different conclusions, suggesting that there were no differences between these post-menopausal populations as long as the non-dairy consumers got enough calcium from other foods and supplements. A recent Mendelian randomization meta-analysis investigating the relationship between the Northern European LCT-13910 C > T gene variant, milk intake, and bone health also reported a lack of associations between the LP mutation and bone mineral density [29]. These researchers also cast doubt on the ability of dietary calcium to improve bone health by itself, and implied that there are additional factors (e.g., protein intake and vitamin D fortification) that need to be considered in relation to dairy intake and bone health.

Recent investigations into the associations between LP status, dairy intake, and health outcomes have provided nuanced findings that are moderated by factors including LP genotype, the type and amount of dairy product consumed, age, gender, body weight, and the specific pathology of the disease being assessed. Additionally, numerous other variables such as mutations in non-LP genes, whole dietary patterns, and environmental factors affect the nutrigenetic relationship between lactase persistence, dairy intake, and health. The science of nutrigenetics is quickly moving beyond the single candidate gene approach, and it is becoming clear that LP mutations are not the only mutations that have the potential to moderate the relationships between dairy intake and disease.

## 3. Metabolic and Hormonal Gene Variants, Dairy Intake, and Health Outcomes

In addition to lactose, dairy products contain a diverse set of constituents (i.e., proteins, fats, vitamins, minerals, and bioactives) that can affect human health. The digestion, metabolism, transport, and excretion of these compounds are all dependent on gene products (i.e., RNAs, and proteins such as enzymes, receptors, peptide hormones, binding proteins, and transport proteins). Therefore, genetic variants that result in altered metabolic and hormonal gene products may influence the relationship between dairy intake and disease risk. The gene variants that have been studied so far in relation to dairy intake include polymorphisms in genes related to lipid metabolism, hormone receptor function, and vitamin D receptor function (Table 2).

### 3.1. Lipid Metabolism SNPs

Saturated fat is one of the most commonly studied dietary compounds in relation to genetic polymorphisms and health, primarily having to do with cholesterol levels and CVD risk [10]. Saturated fat is also one of the main compounds that differ considerably between dairy products, with commonly consumed products ranging from <1% saturated fat (fat-free milk) to over 50% saturated fat (butter). For decades, nutrition studies have provided mixed results regarding the health effects of saturated fats. These inconclusive results have often been attributed to factors such as study design, researcher bias, and the specific type of saturated fats consumed [64]. Until recently, another major factor has often been omitted from these studies, and that is the genetic predispositions of the subjects being studied. The recent advances in nutrigenetic research show that genetic inter-individual variability in how humans respond to dietary fats could help explain why hundreds of past studies have failed to provide a consensus understanding on the effects of saturated fat intake on human health.

A gene-candidate study involving 2000 ethnically diverse participants (Americans of Northern European ancestry and Americans of Puerto Rican origin) was conducted to determine the interactions between an apolipoprotein A2 gene (APOA2) gene variant, dairy product intake, and BMI [10]. In this study, a CC genotype was associated with a higher BMI, but only in those subjects consuming the highest amount of dairy fat. On the other hand, greater dairy fat intake was not associated with a higher BMI in subjects with a CT or TT genotype. Upon further analysis, females with the CC genotype from the American section of the study were also shown to have a higher BMI when consuming the lowest amount of low-fat dairy products. These findings reinforce the idea that the dairy intake and BMI relationship is not solely dependent on any single dietary or genetic factor. Rather, several variables including ethnicity and gender play key roles in how individuals respond to dietary patterns.

A year-long randomized trial of 161 Spanish adults investigated the relationship between dairy fat intake and 14 SNPs within nine different candidate lipid metabolism genes [63]. The participants were randomly assigned to ingest either 500 mL per day of skimmed milk (1 g of saturated fat) daily, or an equivalent amount of semi-skimmed milk (6.7 g of saturated fat) along with their normal diets. The results showed no differences in lipid biomarkers between the different groups even though the groups consumed a difference of more than 5 g/day of saturated dairy fat. When the different SNPs were assessed and corrections were made for multiple testing, only one of the 14 SNPs tested showed an association with dairy fat intake. The TT genotype for peroxisome proliferator-activated receptor alpha (PPARA) rs135549 correlated with reduced total cholesterol/HDL and LDL/HDL-C ratios, while CC and CT genotypes did not. A more recent assessment of dairy intake and candidate SNPs in cholesterol-related genes was performed in 101 Canadian adults [64]. This randomized crossover study assigned participants to consume a prudent diet for four weeks along with either three daily servings of dairy foods (low-fat milk, low-fat yogurt, and cheese) or a non-dairy control matched for total energy but lower in saturated fat. The researchers found multiple SNPs associated with varying lipid responses to the different diets. The GG genotype for the ATP-binding cassette subfamily G, member 5 (ABCG5) rs6720173 was associated with higher total cholesterol and LDL levels compared to CG and CC genotypes. GG and GT genotypes for cholesterol 7α-hydroxylase (CYP7A1) rs3808607 were associated with higher total cholesterol and LDL levels compared to the TT genotype. AA and AG genotypes for 7-dehydrocholesterol reductase (DHCR7) rs760241 were associated with higher LDL levels compared to subjects with the GG genotype. When considered all together, these studies show that individuals with mutations in particular lipid metabolism genes (e.g., APOA2 rs5082, PPARA rs135549, ABCG5 rs6720173, CYP7A1 rs3808607, DHCR7 rs760241) may show differential sensitivity to the health effects of dairy food intake, especially when it comes to the intake of products with different levels of saturated fats.

### 3.2. Hormone and Hormone Receptor SNPs

In addition to lipid metabolism SNPs, hormone and hormone receptor SNPs have also been studied in the context of dairy intake and health outcomes. Researchers from the InterAct Consortium investigated the relationships between incretin hormone SNPs, whey-containing dairy intake, and risk for T2D in 18,638 European adults [70]. Whey-containing dairy foods were chosen for this analysis since whey protein has been shown to influence the secretion of the incretins glucagon-like peptide-1 (GLP-1) and glucose-dependent insulinotropic peptide (GIP) to a greater degree than other protein sources. After more than 12 years of follow-up, the researchers found no associations between any of the seven incretin gene variants, whey-containing dairy servings, and T2D risk. A genetic risk score assessment was conducted on the participants from this study and also showed no interactions between the incretin gene variants, whey-containing dairy intake, and T2D risk. In simpler terms, a single serving per day of whey-containing dairy foods did not affect T2D risk even in the subjects who had the highest number of incretin-related allele SNPs.

A gene-candidate study involving 945 European adults at high risk for CVD from the PREDIMED-Valencia Study was conducted to determine if a particular genetic polymorphism in the somatostatin receptor 2 (SSTR2) gene interacts with dietary or anthropometric variables [69]. Among other functions, the somatostatin system (which includes its receptors) can inhibit the secretion of growth hormone, gut hormones, and pancreatic hormones; affecting cell growth, pituitary gland function, body weight regulation, and neuro-endocrine function [69,71]. The PREDIMED-Valencia study results showed that the rs1466113 C > G polymorphism tracked with both dairy intake and obesity. Individuals with one or more dominant G alleles tended to have a higher BMI and consumed a greater amount of dairy, while those with the CC genotype had a lower BMI, a lower odds ratio for obesity, and consumed a smaller amount of dairy. In addition to consuming less dairy, the CC group also consumed less total protein, meat, and legumes on a daily basis, suggesting that this particular gene might be associated with regulating protein intake, and that this particular SNP could influence several aspects of dietary intake and body weight beyond that of dairy consumption. 

### 3.3. Vitamin D Receptor SNPs

Studies on vitamin D receptor (VDR) polymorphisms and dairy intake have provided key insights into how different polymorphisms in the same gene interact with diet to influence health outcomes. Indeed, while one VDR polymorphism might affect receptor-binding activity, another might pertain to receptor binding strength or substrate affinity. These differences can affect how the receptors will interact with dietary constituents, and in turn will affect how those dietary constituents are absorbed, transported, metabolized, and excreted.

A 12-week long intervention study investigated the relationships between VDR FokI polymorphism, vitamin D fortified dairy intake, and inflammatory and antioxidative markers in 140 Iranian subjects with T2D [66,67]. Subjects were assigned to ingest either 500 mL of vitamin D fortified yogurt per day (1000 IU of vitamin D), or a control yogurt beverage that was not fortified with vitamin D. The subjects who consumed the fortified yogurt had higher vitamin D levels and lower inflammatory markers (high-sensitivity C-reactive protein, interleukin-4, and interleukin-6) [66], and higher antioxidative markers (glutathione and total antioxidant capacity) [67], compared to subjects who consumed the plain yogurt. When genotype was assessed, subjects with the FF genotype had higher vitamin D levels than the ff genotype, and those with a FF genotype also had the greatest reduction in high-sensitivity C-reactive protein and interleukin-6 compared to both the Ff and ff genotypes. These findings suggest that FokI gene variants may influence the ability to utilize vitamin D from dairy foods and could thereby ameliorate the inflammatory response in diabetic subjects.

A similar 12-week long study was conducted by the same researchers to determine the relationships between a different VDR polymorphism (Cdx2 polymorphism), vitamin D fortified yogurt intake, and central obesity in 60 Iranian subjects with T2D [68]. The subjects were randomly assigned to consume either 500 mL of 1000 IU vitamin D fortified yogurt per day or the same amount of an unfortified control yogurt. At the end of the study, all subjects who consumed the fortified yogurt had higher vitamin D levels and reduced central adiposity measures compared to those who consumed the unfortified yogurt. When the Cdx2 genotype (AA, AG, GG) was assessed, circulating vitamin D levels were increased and central adiposity measures were decreased to a greater degree in the AA group, compared to the G-allele carriers. Overall, these results suggest that vitamin D fortification improves the health benefits of dairy products when consumed by type 2 diabetic subjects, but these effects appear to be moderated by specific genetic variants in the vitamin D receptors.

When multiple VDR polymorphism (FokI, Cdx2, ApaI, BsmI, TaqI) and dairy intake were investigated in relation to colorectal cancer recurrence in 480 subjects from the United Kingdom [65], only certain Apal genotypes appeared to interact with dairy intake and health outcomes. The AA and Aa Apal genotypes interacted with higher dairy product consumption to reduce colorectal cancer recurrence, but the aa genotype did not. Many of dairy’s effects on colorectal cancer recurrence in this study were dependent on the type of dairy product consumed, with milk being more protective than other dairy products. Interestingly, these effects also appear limited to dairy-derived vitamin D, with intake of vitamin D from food and/or supplements not showing the same beneficial influence on colorectal cancer recurrence reduction.

In combination with the findings from LP and dairy intake studies, studies on non-LP gene variants and dairy intake provide convincing evidence that an array of gene variants are responsible for significant interindividual variations in dietary intake patterns and metabolic responses. Many of these gene–diet interactions converge to influence body weight, cardiometabolic disease risks, cancer risk and bone health. Further findings from the investigations of gene–dairy–disease interactions will contribute greatly to the development of more genetically informed and personalized recommendations regarding dairy product intake and disease risk management.

## 4. Conclusions

A major aim of personalized nutrition is to turn a person’s nutrigenetic and nutrigenomic information into highly specific dietary advice that can be used to improve or maintain health. This type of dietary advice has the potential to revolutionize the fields of nutrition and healthcare. However, at present, the science of personalized nutrition is just scratching the surface of its potential, and we must be careful not to label it a panacea before proving its efficacy. While this review of nutrigenetic studies has focused solely on one food group and a few select genetic mutations, it has clearly shown the complexities of understanding and utilizing the information gathered from studies of gene–diet interactions. Nutrigenetic studies that have included data on dairy intake provide mixed results on health outcomes. Many of these studies show that LP is associated with higher dairy intake and BMI, but they also tend to show that LP and higher dairy intake are not consistently associated with cardiometabolic disease risk, certain types of cancer occurrence, or bone health. Nutrigenetic studies investigating the effects of polymorphisms in genes related to lipid metabolism, hormone receptor function, and vitamin D receptor function also show mixed results and the potential for differential sensitivity between genotypes to the health effects of dairy food intake. More research is necessary on polygenic and multifactorial relationships in the context of diet and disease. In the end, these nutrigenetic relationships are likely dependent on many other factors besides the individual SNPs tested, such as the type and amount of dairy product consumed, gender, age, ethnicity, and other genetic mutations that affect the metabolism, transport, or storage of nutrients in the body.

The interindividual variation between humans is on the magnitude of millions of SNPs, so the insights gained from a limited focus on a few SNPs having to do with dairy intake reveal only a small part of a person’s nutrigenetic story. Furthermore, additional research is needed on the relationships between different gene variants and their abilities to interact and influence each other in antagonistic, additive, or synergistic ways. In summary, nutrigenetic research which is focused on the relationships between single SNPs (of which there are millions) and single food groups (which may be comprised of a diverse array of products with varying combinations and amounts of nutrients and bioactives), may provide great insights into improving the science of personalized nutrition. However, this type of research is only part of the personalized nutrition equation. Nutrigenomic responses (epigenomics, transcriptomics, proteomics, metabolomics) to different types and amounts of dairy products, along with microbiome data, and phenotypic/characteristic traits (age, gender, activity level, disease status, etc.) must also be accounted for, since these factors can all interact with the diet to influence health.

## Figures and Tables

**Table 1:** Nutrigenetic studies on lactase persistence, dairy intake, and health outcomes in adults.

Reference	Study Design	Population	Variables	Outcomes
**Obesity and Cardiometabolic Disease**				
Lehtimäki et al., 2008. [15]	Observational, longitudinal—participants followed for an average of 21 years	2109 young and healthy adults from Finland	Milk and Dairy Intake Carotid intima-media thickness, carotid artery compliance, brachial artery flow-mediated dilation	No significant association between LP and carotid intima-media thickness, carotid artery compliance or brachial artery flow-mediated dilation were found after adjustment for the use dairy products.
Almon et al., 2012. [16]	Meta-analysis, observational, cross-sectional	551 adults of European descent from the Canary Islands Nutrition Survey (ENCA) in Spain	Milk Intake BMI, obesity	LP was associated with higher BMI, while lactase non-persistence was not after adjustment for milk intake.
Almon et al., 2010. [17]	Meta-analysis, observational, cross-sectional	551 adults of European descent from the Canary Islands Nutrition Survey (ENCA) in Spain	Milk Intake Metabolic Syndrome risk markers	LP was associated with a higher odds ratio for metabolic syndrome than lactase non-persistence after adjustment for milk intake. This relationship was stronger for women than men.
Corella et al., 2011. [18]	Observational, cross-sectional	940 elderly Spanish adults with high risk for CVD	Milk and Dairy Intake BMI, obesity	LP was associated with obesity risk. These associations were found to be significant only among those consuming moderate or high lactose intakes.
Lamri et al., 2013. [19]	Observational, longitudinal—participants followed for an average of 9 years	3575 Caucasians born in mainland France	Dairy Intake Metabolic Syndrome risk markers	LP was associated with higher BMI mainly in those consuming high amounts of dairy products. LP was associated with higher risk for metabolic syndrome, but this association disappeared after adjustment for BMI.
Bergholdt et al., 2015. [20]	Observational, cross-sectional and longitudinal—participants followed for an average of 5.5 years	97,811 adults from the Danish general population	Milk Intake Overweight, obesity, T2D risk	High milk intake was not associated with risk of T2D or overweight-obesity, observationally or genetically via LP.
Bergholdt et al., 2015. [21]	Observational, cross-sectional and longitudinal—participants followed for an average of 5.4 years	98,529 adults of Danish descent	Milk Intake Ischemic heart disease, myocardial infarction	LP was not associated with plasma levels of total cholesterol, low-density lipoprotein cholesterol, high-density lipoprotein cholesterol, triglycerides or glucose, nor with blood pressure. Milk intake was not associated with risk of ischemic heart disease or myocardial infarction, observationally or genetically.
Hartwig et al., 2016. [22]	Meta-analysis, observational, longitudinal—participants followed for an average of 30 years	2843 adults from the 1982 Pelotas (Southern Brazil) Birth Cohort	Milk Intake BMI, obesity, systolic and diastolic blood pressure	LP was associated with higher BMI and higher odds of overweight-obesity. Milk intake was not consistently associated with changes in blood pressure.
Smith et al., 2016. [23]	Meta-analysis, multiple study designs included	20,089 adults of American (Hispanics, African-Americans and Whites) and Mediterranean descent	Milk Intake CVD biomarkers, mortality	Milk intake was not associated with CVD biomarkers, CVD or mortality either generally or in sub-groups. Lactase persistence was inconsistently associated with glucose and lipids, and not associated with CVD or total mortality in the whole population. LP was associated with higher CVD and mortality risk in women but not in men.
Ding et al., 2017. [24]	Meta-analysis, multiple study designs included	197,322 adults from Europe, the US, and Australia	Dairy Intake Systolic blood pressure, hypertension	LP was not associated with systolic blood pressure or risk of hypertension. No associations were found between dairy intake and blood pressure.
**Colorectal, Prostate, and Renal Cell Cancer**				
Szilagyi et al., 2006. [25]	Meta-analysis, observational, cohort and case-control	Data from 80 studies (27 cohort and 53 case-control reports)	Dairy Intake Colorectal cancer	The highest level of dairy food consumption protects subjects in both high and low lactase non-persistence regions, but not in regions with significant mixed LP and non-persistent populations.
Torniainen et al., 2007. [12]	Observational, case-control	LP study: 4153 Finnish and Swedish patients and 2315 controls. Milk intake study: 1499 Swedish prostate cancer patients and 1130 controls	Milk Intake Prostate cancer	LP showed no association with prostate cancer risk. High intake of low-fat milk was associated with a significantly increased relative risk of prostate cancer, whereas no association was observed between dietary intakes of total milk, high-fat milk, all dairy products, or dairy products high or low in lactose and risk of prostate cancer.
Timpson et al., 2010. [26]	Observational, case-control	915 cases and 2346 controls from adults of Eastern Europe and Russian descent	Milk Intake Renal cell carcinoma	In cancer cases, LP was associated with higher milk intake, but was not associated with renal cell carcinoma. In controls, milk consumption was associated with confounding factors, including smoking and education.
Travis et al., 2013. [13]	Observational, case-control	630 European men with prostate cancer and 873 matched controls	Milk and Dairy Intake Prostate cancer	LP was associated with greater milk intake, but was not significantly associated with prostate cancer risk.
**Bone Density, Fractures, and Osteoporosis**				
Obermayer-Pietsch et al., 2004. [27]	Observational, cross-sectional	258 postmenopausal women from Austria	Milk Intake Bone mineral density and bone fractures	LP was associated with higher milk intake, fewer bone fractures, and higher bone mineral density at the hip and the lumbar spine compared to lactase non-persistence.
Enattah et al., 2005. [28]	Observational, cross-sectional, case-control	453 elderly Finnish women. 52 elderly Finnish women with osteoporotic fractures. 59 controls without osteoporosis	Dairy Intake Osteoporosis, bone mineral density, and fractures	Lactose mal-digestion and lactose intolerance were not risk factors for osteoporosis, if calcium intake from diet and/or supplements remained sufficient.
Yang et al., 2017. [29]	Meta-analysis, observational, mixed design	Five studies of 102,750 adults of mixed descent	Milk and Dairy Intake Osteoporosis, ischemic heart disease, T2D	LP and milk consumption were not clearly associated with bone mineral density, ischemic heart disease, or T2D.

Abbreviations: Lactase Persistence (LP), Cardiovascular Disease (CVD), Type 2 Diabetes (T2D), Body Mass Index (BMI).

**Table 2:** Nutrigenetic studies on non-lactase polymorphisms, dairy intake, and health outcomes in adults.

Reference	Study Design	Population	Gene Variants	Variables	Outcomes
**Lipid Metabolism SNPs**					
Smith CE et al., 2013. [10]	Observational, cross-sectional	955 adults from the Boston Puerto Rican Health Study and 1116 adults from the Genetics of Lipid Lowering Drugs and Diet Network study	APOA2-265 T > C (rs5082)	Total dairy, higher-fat dairy (>1%), and low-fat dairy (≤1%) BMI, Body Weight	There was a significant interaction between the APOA2-265 T > C polymorphism and dairy food intake. Individuals with the CC genotype who consumed more higher-fat dairy products had a higher BMI compared with those consuming less higher-fat dairy products.
Loria-Kohen et al., 2014. [63]	Intervention, randomized trial (1 year)	161 middle-aged Spanish adults	14 SNPs in 9 genes related to lipid metabolism were examined	500 mL per day of skimmed or semi-skimmed milk for 1 year in addition to their usual diets. CVD risk markers	A TT genotype for PPARA rs135549 was associated with a reduction in the total cholesterol/HDL and LDL/HDL ratios after 1 year of skimmed milk intake. No differences were observed after consuming either skimmed or semi-skimmed milk in the C allele carriers.
Abdullah et al., 2016. [64]	Intervention, RCT (4 weeks)	101 middle-aged Canadian adults	ABCG5, CYP7A1, DHCR7	3 servings per day of dairy or energy-matched control on background of a prudent diet. Serum lipids	Genetic variations in ABCG5, CYP7A1, and DHCR7 may contribute to differing responses of serum cholesterol to dairy intake among healthy adults.
**Vitamin D Receptor SNPs**					
Hubner et al., 2008. [65]	Sub-study of an RCT using aspirin and folate for the prevention of colorectal adenoma recurrence	480 participants in the United Kingdom Colorectal Adenoma Prevention trial	Cdx2, FokI, BsmI, ApaI and TaqI	Milk and Dairy Product Intake Colorectal cancer	VDR polymorphism genotypes and haplotypes did not directly alter colorectal cancer recurrence risk, but the reduction in risk associated with high dairy product intake was confined to individuals with ApaI aA/AA genotype.
Neyestani et al., 2013. [66]	Intervention, RCT (12 weeks)	140 middle-aged Iranian adults with T2D	FokI	500 mL yogurt drink (doogh) per day fortified with 1000 IU vitamin D T2D; glycemic status, lipid profiles, inflammatory biomarkers	The FF genotype group had the largest decrease of C-reactive protein and interleukin-6 compared with the Ff and ff groups. The vitamin D response of the ff genotype group was the lowest after consuming the vitamin D fortified doogh.
Shab-Bidar et al., 2015. [67]	Intervention, RCT (12 weeks)	140 middle-aged Iranian adults with T2D	FokI	500 mL yogurt drink (doogh) per day fortified with 1000 IU vitamin D or control yogurt drink with no vitamin D Oxidative stress biomarkers	No significant association between FokI genotypes and oxidative stress biomarkers, but the the ff variant subgroup showed the weakest response to vitamin D fortified doogh.
Shab-Bidar et al., 2015. [68]	Intervention, RCT (12 weeks)	60 middle-aged Iranian adults with T2D	Cdx2	500 mL yogurt drink (doogh) per day fortified with 1000 IU vitamin D or control yogurt drink with no vitamin D T2D, glycemic and adiposity biomarkers	Daily intake of vitamin D fortified doogh for 12 weeks improved the central obesity indices in T2D subjects, and the improvement was more pronounced in the carriers of the AA genotype of VDR-Cdx2.
**Hormone and Hormone Receptor SNPs**					
Sotos-Prieto et al., 2010. [69]	Observational, cross-sectional	945 high-CVD risk older subjects participating in the PREDIMED–Valencia Study	rs1466113 G > C in SSTR2	All food groups, including dairy BMI, obesity	Homozygous subjects for the C allele had significantly lower BMI and odds ratio for obesity than G-allele carriers. There were also significant differences in dairy product and protein intakes between CC- and G-allele carriers.
InterAct Consortium 2016. [70]	Observational, case-cohort, average follow up of 12.5 years	18,638 middle-aged, normal weight adults from EPIC-InterAct study	TCF7L2 rs12255372, TCF7L2 rs7903146, KCNQ1 rs163171, KCNQ1 rs163184, KCNQ1 rs2237892, GIPR rs10423928, WFS1 rs10010131	Intake of whey- containing dairy products T2D risk, incretins	No significant differences between any of the possible genotypes investigated and risk of T2D per one serving per day increment of Whey-containing dairy (150 g/day).

Abbreviations: Single Nucleotide Polymorphism (SNP), Randomized Controlled Trial (RCT), Body Mass Index (BMI), Cardiovascular Disease (CVD), Type 2 Diabetes (T2D), High-Density Lipoprotein (HDL), Low-Density Lipoprotein (LDL), Vitamin D Receptor (VDR), Somatostatin Receptor 2 (SSTR2), Peroxisome Proliferator-Activated Receptor Alpha (PPARA), Apolipoprotein A2 (APOA2), Cholesterol 7α-Hydroxylase (CYP7A1), ATP-Binding Cassette Subfamily G, Member 5 (ABCG5), 7-Dehydrocholesterol Reductase (DHCR7),Transcription Factor 7-like 2 (TCF7L2), Gastric Inhibitory Polypeptide Receptor (GIPR), Potassium Voltage-Gated Channel Subfamily Q Member 1 (KCNQ1), Wolframin ER Transmembrane Glycoprotein (WFS1).

## Abbreviation

LCT	Lactase Gene
LP	Lactase Persistence
SNP	Single Nucleotide Polymorphism
PKU	Phenylketonuria
MTHFR	5,10-Methylene Tetrahydrofolate Reductase
BMI	Body Mass Index
CVD	Cardiovascular Disease
T2DM	Type 2 Diabetes Mellitus
BMD	Bone Mineral Density
VDR	Vitamin D Receptor
APOA2	Apolipoprotein A2
ABCG5	ATP-Binding Cassette Subfamily G, Member 5
CYP7A1	Cholesterol 7α-Hydroxylase
DHCR7	7-Dehydrocholesterol Reductase
PPARA	Peroxisome Proliferator-Activated Receptor Alpha
SSTR2	Somatostatin Receptor 2
GLP-1	Glucagon-Like Peptide-1
GIP	Glucose-Dependent Insulinotropic Peptide
